# Supplementary material for: Rescue of Infectious Sindbis Virus by Yeast Spheroplast-Mammalian Cell Fusion
Source: Viruses. 2021 Apr 1;13(4):603. doi: 10.3390/v13040603 (PMC8066160; doi:10.3390/v13040603)
Supplement: Supplementary file 1 [file viruses-13-00603-s001.pdf]

Figure S1

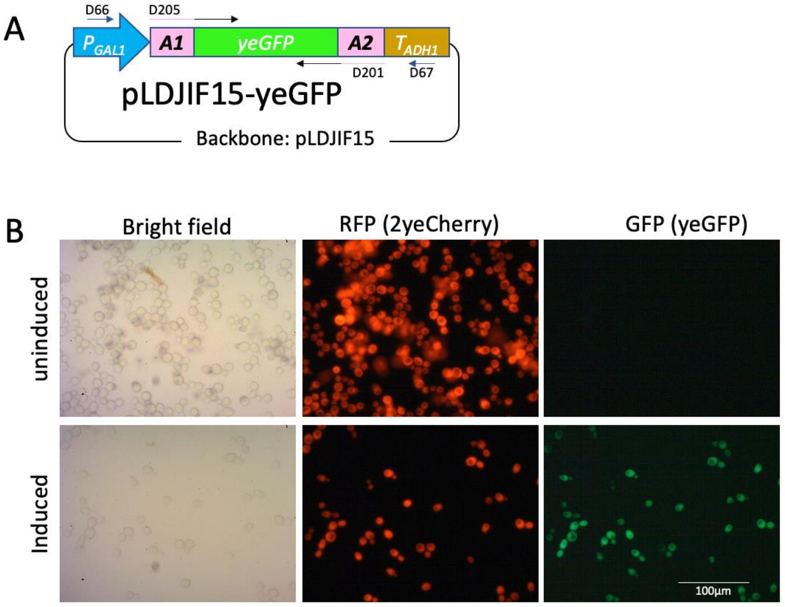

Figure S1. pLDJIF15 is a vector for galactose induced protein expression. (**A**.) A schematic of pLDJIF15-yeGFP. Not drawn to scale. A codon-optimized GFP for yeast expression (yeGFP) was amplified with primers D205 and D201 and inserted between Adapter 1 and 2 using Gibson Assembly. See GenBank files in supplementary data for sequences. (**B)**. Fluorescence micrographs of fusion experiments. yLDJIF22 (W303α transformed with pLDJIF15-yeGFP) was grown overnight in –TRP medium was used to inoculate –TRP medium (uninduced) or YPG medium (induced) for 5 hours at 30°C.

Figure S2

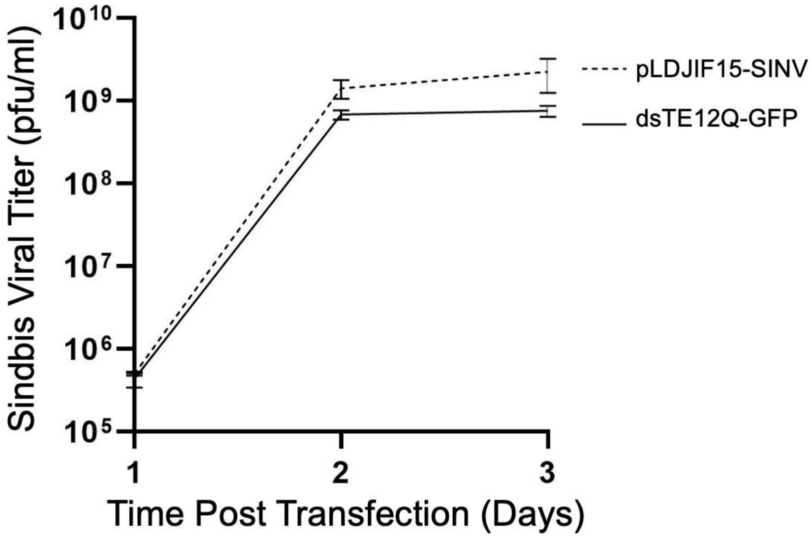

Figure S2. pLDJIF15-SINV and dsTE12Q-GFP rescue Sindbis viral particles via transfection of plasmid DNA. dsTE12Q-GFP is the Parental SINV cDNA construct
